# Yellow Fever

**Published:** 1893-09

**Authors:** 


### *EDITORIAL.*

The office of THE JOURNAL is in room 330 Equitable Building.
Address all communications, and make all remittances payable to THE ATLANTA MEDICAL AND SURGICAL JOURNAL, Atlanta, Ga.
Articles for publication should reach this office not later than the 15th instant.
The editors are not responsible for views expressed by contributors.

### YELLOW FEVER.

Yellow fever has invaded the port of Brunswick. As is always the case in the beginning of such invasion, the city, and a certain number of the doctors of the afflicted community, hope against hope that it is not yellow fever, and that it is certainly not epidemic. But the clinical history, and Guiteras, say it is yellow fever and that the port is infected.

Two facts stand out more prominently than all others in connection with these sad developments. First, the lack of sympathy and hearty co-operation with the government experts on the part of the people afflicted. Second, the lack of strict quarantine by the municipal government, which evidently was the cause of the invasion.

The question of the propriety of the general government taking absolute control of port quarantine or of modified and co-operative quarantine seems, in the case of Brunswick, to have given us an object lesson fully illustrating the fact that the best results can only be expected where we have the dicta of an expert executed by a broad-minded, sympathetic and *locally well established* set of officers who not only have the fullest confidence of their people, but who also have, beyond any question, the good of the community at heart, and the courage to carry out their convictions.

Naturally, people are jealous of their property rights, and reluctantly surrender them to officers for destruction, especially if they regard the officer as an alien to the community which he is appointed to command. Therefore, it is important that we should have a combined municipal, State and national quarantine.

The sacrifice that Brunswick has made to ignorance and carelessness, life and treasure, can in no way be charged to any other account. Life cannot be redeemed, and years of prosperity will be required to restore the actual loss of values already acquired, without reference to loss of prospective increase based upon the reasonable expectation of accretion about a favorable nucleus.

Shutting our eyes to the picture of horror and desolation, closing our ears to the wailing of the bereaved, and turning from the appeals of those who are without food and shelter, we cannot but see that Georgia, without the inspiration of love or charity, would have made a profitable investment by sustaining her State Board of Health, even at the cost of many millions.

No part of this writing is intended as a criticism of the individuals who have had the responsibility and burden and thankless task of doing, under such adverse circumstances and disheartening question, the work of heroes.

But the State government should provide such facilities for diagnosing and controlling diseases of this type, that no city or community should pay the forfeit of its life for the love of a friend

The yellow fever germ has been discovered, is known to every microscopist, and while the gross symptoms of some types of other fever may be similar or identical, there is no reason why a State so rich, powerful and extensive in her territory as Georgia should allow an enemy so dreaded and so deadly to take possession of one of her finest ports.

How well those in charge at Brunswick have performed their duty can be judged when we contemplate the fact that in a city of fifteen thousand, where all the indications were that an epidemic was imminent, only two cases have developed under their management.

The methods, isolation and depopulation, put into practice for stopping the fever, whatever of criticism they may have brought to the officers, have been efficacious.

The town is infected, and if temporarily depopulating the place is to be deplored because of the inconvenience and expense thereof, what is it in comparison with a permanent depopulation?

The lesson is a terrible one to Brunswick, Georgia and the whole country.

Let us profit by it, and let every man that can see how this pestilence could have been averted bring his influence to the establishing of such regulations by law as will make definite the responsibility of him who dares to trifle with this arch enemy of man.

In papers read last April before the State Medical Association of Georgia urging its action towards securing a State Board of Health for Georgia His Excellency Governor Northen was quoted as follows from his message to the last General Assembly:

"We may any year be subjected to the invasion of yellow fever from the Spanish countries to the south of us, or of the Asiatic cholera through the northern ports. We are now utterly helpless to deal with these plagues, and should either menace us we should have to appeal again to the Federal Government. Beside these dangers from without we have many sources of danger within our borders, and an efficient board of health would be a constant safeguard to the health of our people and afford a feeling of security gainst epidemics and disease."

Only too soon have his prophetic words been fulfilled.

